# Synthesis of Pyrrolo[3,4-*b*]pyridin-5-ones via Multicomponent Reactions and In Vitro–In Silico Studies Against SiHa, HeLa, and CaSki Human Cervical Carcinoma Cell Lines

**DOI:** 10.3390/molecules24142648

**Published:** 2019-07-22

**Authors:** Daniel Segura-Olvera, Ailyn N. García-González, Ivette Morales-Salazar, Alejandro Islas-Jácome, Yareli Rojas-Aguirre, Ilich A. Ibarra, Erik Díaz-Cervantes, Sofía Lizeth Alcaraz-Estrada, Eduardo González-Zamora

**Affiliations:** 1 Departamento de Química, Universidad Autónoma Metropolitana-Iztapalapa, San Rafael Atlixco 186, Col. Vicentina, C.P. 09340, Iztapalapa, Mexico City, Mexico; 2 Departamento de Polímeros, Instituto de Investigaciones en Materiales, Universidad Nacional Autónoma de México, Circuito Exterior S/N, Ciudad Universitaria, Coyoacán, Mexico City C.P. 04510, Mexico; 3 Laboratorio de Fisicoquímica y Reactividad de Superficies, Instituto de Investigaciones en Materiales, Universidad Nacional Autónoma de México, Circuito Exterior S/N, Ciudad Universitaria, Coyoacán, Mexico City C.P. 04510, Mexico; 4 Departamento de Alimentos, Centro Interdisciplinario del Noreste, Universidad de Guanajuato, Tierra Blanca, Guanajuato C.P. 37975, Mexico; 5 División de Medicina Genómica, Centro Médico Nacional 20 de Noviembre, ISSSTE, Félix Cuevas 540, Col. Del Valle Sur, Benito Juárez, Mexico City C.P. 03100, Mexico

**Keywords:** pyrrolo[3,4-*b*]pyridin-5-ones, paclitaxel, αβ-tubulin, cervical cancer, SiHa, HeLa, Caski, molecular docking, QSAR, pharmacophore model

## Abstract

A series of 12 polysubstituted pyrrolo[3,4-*b*]pyridin-5-ones were synthesized via a one-pot cascade process (Ugi–3CR/*aza* Diels-Alder/*N*-acylation/decarboxylation/dehydration) and studied in vitro using human epithelial cervical carcinoma SiHa, HeLa, and CaSki cell line cultures. Three compounds of the series exhibited significative cytotoxicity against the three cell lines, with HeLa being the most sensitive one. Then, based on these results, in silico studies by docking techniques were performed using Paclitaxel as a reference and αβ-tubulin as the selected biological target. Worth highlighting is that strong hydrophobic interactions were observed between the three active molecules and the reference drug Paclitaxel, to the αβ-tubulin. In consequence, it was determined that hydrophobic–aromatic moieties of bioactive compounds and Paclitaxel play a key role in making stronger interactions to the ligand–target complex. A quantitative structure activity relationship (QSAR) study revealed that the six membered rings are the most significant molecular frameworks, being present in all proposed models for the in vitro-studied cell lines. Finally, also from the docking interpretation, a ligand-based pharmacophore model is proposed in order to find further potential polyheterocyclic candidates to bind stronger to the αβ-tubulin.

## 1. Introduction

Cancer is a disease currently killing 13% of the population worldwide mainly because patients do not receive efficient treatments. With reference to the incidence of cancer, it has been estimated that by 2025, there will be approximately 20 million new cancer patients worldwide per year [1]. Cancer appears when cells of certain tissues avoid apoptosis (natural process of programmed cell death) due to various factors, the most important being damage in genetic material [2]. There are more than 200 known types of cancer; some show faster proliferative activity than others [3]. For example, cervical cancer (caused by papillomaviruses, i.e., genotypes HPV-16 and HPV-18) is one of the most aggressive among all [4]. For this reason, it has been widely studied in vitro using human epithelial cervical carcinoma cell line cultures SiHa [5], HeLa [6], and CaSki [7]. In addition, within the spectrum of anticancer compounds, polyheterocycles play a central role due to their interesting pharmacophoric, pharmacodynamic, and pharmacokinetic properties [8], despite the fact that they often do not obey rules and parameters related to the applicability of big/complex organic molecules like most drugs, e.g., the Lipinski rules [9], Ghose rules [10], and Veber criteria [11].

Multicomponent reactions (MCRs) are one-pot processes, in which at least three easily accessible or commercially available reagents are combined sequentially to form complex products, where most of the atoms come from the starting reagents [12,13]. Moreover, MCRs have been used successfully to synthesize novel drug-like anticancer compounds (including polyheterocycles) [14] with different associated and specific action mechanisms; for instance, by inhibition of (i) tubulin polymerization; (ii) kinesins; (iii) kinases; (iv) multidomain extra-terminal (MET) proteins; (v) p53 activity; (vi) transcription initiation factor (TIF) proteins; or vii) topoisomerases (TOP), just to highlight a few [15]. In this context, one of the most important biological targets in the fight against cancer are the tubulins, which belong to microtubules (specifically the α and the β) involved in mitosis processes [16,17,18]. Thus, the present work describes the use of pyrrolo[3,4-*b*]pyridin-5-ones (reported or new) against SiHa, HeLa, and CaSki human cervical carcinoma cell cultures, as well as in silico studies through molecular docking techniques to propose a ligand-based pharmacophore model, in addition to a QSAR study. For the latter, αβ-tubulin was used as the biological target. α- and β-tubulins polymerize into microtubules, structures involved in cell division. The increase in cell division, being the main feature in cancer malignancies, is a highly attractive target for medicinal/synthetic chemists and hence, the design and synthesis of compounds interfering in microtubule dynamics, by means of tubulin-binding, is still being of great importance, as well as in vitro and in silico activity evaluation [19,20].

## 2. Results and Discussion

### 2.1. Synthesis

**Figure 1:**
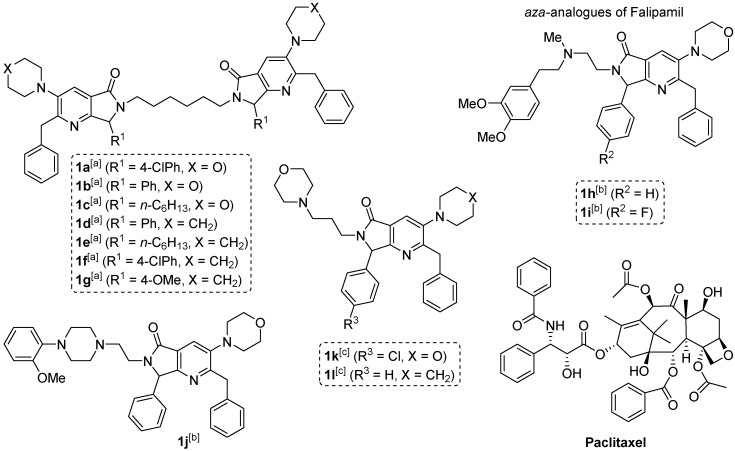
Initially tested pyrrolo[3,4-*b*]pyridin-5-ones. [a] Synthesis reported in Reference [32]. [b] Synthesis reported in Reference [22]. [c] New pyrrolo[3,4-*b*]pyridin-5-ones. The products were synthesized and used for the in vitro studies as racemic mixtures.

**Scheme 1:**
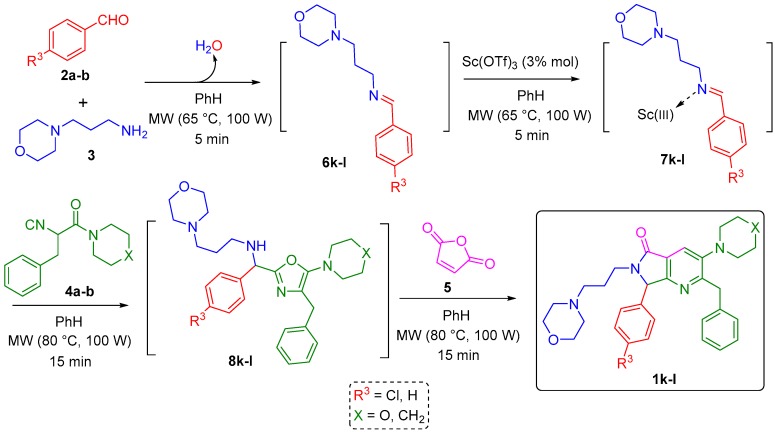
Synthesis of the new pyrrolo[3,4-b]pyridin-5-ones **1k**–**l**.

In our research group, we hold an ongoing program to develop short and versatile MCR-based strategies toward novel and complex polyheterocycles of interest in various fields, like optics [21] and medicinal chemistry [22]. One polyheterocyclic system that we can synthesize à la carte is the pyrrolo[3,4-*b*]pyridin-5-one [23], which is considered as a privileged aza-analogue of the isoindolin-1-one [24], the latter being the structural core of various natural and synthetic anticancer agents [25]. In this context, stepwise methodologies have been reported in synthesizing novel pyrrolo[3,4-*b*]pyridin-5-ones, for example, Devasthale and co-workers synthesized a panel of pyrrolo[3,4-*b*]pyridin-5-ones, evaluating in vitro their properties as inhibitors of the dipeptidyldipentidase-4 (DPP-4) [26]. However, one efficient, versatile, and robust method to construct this polyheterocyclic core is through a one-pot cascade sequence Ugi–3CR/*aza* Diels–Alder cycloaddition/*N*–acylation/decarboxylation/dehydration, reported first by Zhu and co-workers [27], and further optimized by us many times [22,23,28,29,30,31,32,33,34]. Thus, for the in vitro assays against SiHa, HeLa, and CaSki cell lines, we synthesized some of our previously reported pyrrolo[3,4-*b*]pyridin-5-ones **1a**–**j** [22,32], and the new ones **1k**–**l**, specifically prepared for the present study (Figure 1). Thus, 4-chlorobenzaldehyde (**2a**) or benzaldehyde (**2b**) were combined sequentially with the 3-morpholinopropan-1-amine (**3**), the corresponding isocyanides **4a**–**b**, and maleic anhydride (**5**) via the strategy mentioned before, using benzene as solvent, scandium (III) triflate as Lewis-acid catalyst, and microwaves (MW) as heat source to afford the polyheterocycles **1k**–**l** in 46% (**1k**) and 45% (**1l**) yields, respectively, which are excellent considering the molecular complexity of the products, and that only one experimental procedure was needed in their synthesis (Scheme 1). The intermediates **6k**–**l**, **7k**–**l**, and **8k**–**l** were not isolated.

It is worth noting that the newly synthesized products **1k**–**l** were fully characterized by their physicochemical properties, as well as by the classical spectroscopic techniques ^1^H and ^13^C NMR, IR, and HRMS (See the supporting information for further details). In the same context, samples of compounds with purity > 99% (assessed by ^1^H NMR) were used for the in vitro studies.

### 2.2. In Vitro Studies

SiHa, HeLa, and CaSki cells have been used as cellular models for cervical carcinoma because endogenous HPV genome remains stable through repeated passages. It has been reported that each cell line responds quite differently to treatments with chemical agents that induce cell death [35,36,37]. The latter is due to the different origin and properties of the cell lines. HeLa cell line was obtained from a cervical adenocarcinoma of an Afromerican female and has 10 to 50 copies of HPV-18. SiHa and CaSki have HPV-16, but CaSki was isolated from a cervical carcinoma small bowel metastasis and has 60 to 600 copies of the virus. Moreover, SiHa is from a grade II squamous cell carcinoma with only 1 to 2 copies of the virus [38]. It is worth mentioning that SiHa and CaSki were used as models for studying resistance and sensitivity to treatment [39].

**Figure 2:**
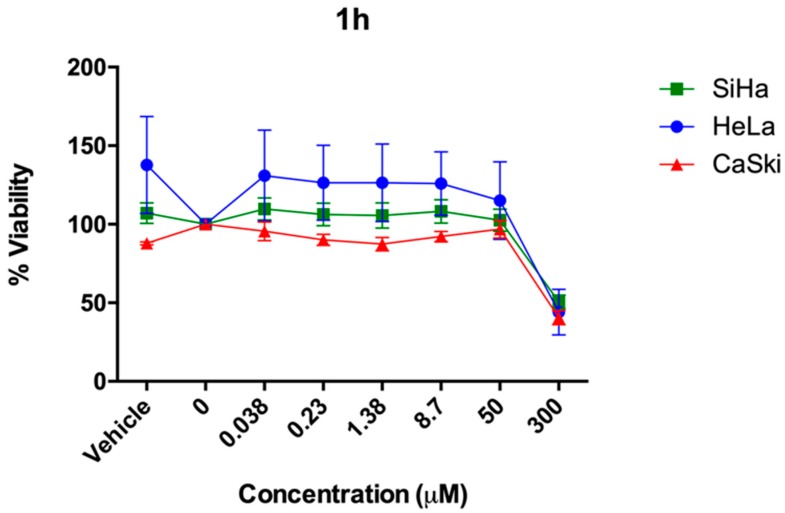
Cell viability of **1h** on human cervical cancer cells SiHa, HeLa, and CaSki.

The cytotoxic effect of each compound against cervical cancer cells was determined using an MTS assay. Figure 2 shows that all cell lines were similarly affected by the compound **1h** in concentrations above 50 μM with the antiproliferative effect, being much more evident at 300 μM concentration, at which viability decreased to 50.58 ± 4.33% for SiHa, 44.04 ± 14.45% for HeLa, and 40.57 ± 4.54% for CaSki.

**Figure 3:**
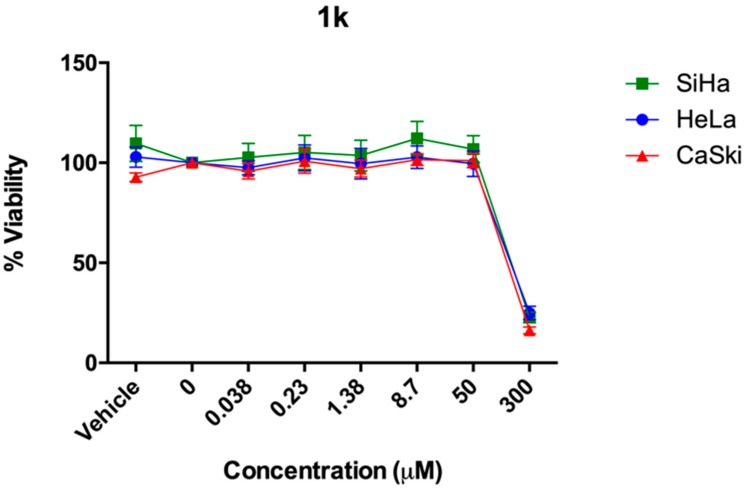
Cell viability of **1k** on human cervical cancer cells SiHa, HeLa, and CaSki.

There was not significant cytotoxicity of **1k** at concentrations below 50 µM. However, it is notable that at 300 µM, **1k** reduced cell survival in much higher extent than **1h**. SiHa and HeLa cells were affected in a similar manner with 22.7 ± 2.6% and 24.9 ± 3.38% viability values, respectively. On the other hand, CaSki displayed a significant sensitivity, as only 16.2 ± 1.7% of the cells remained viable (Figure 3).

**Figure 4:**
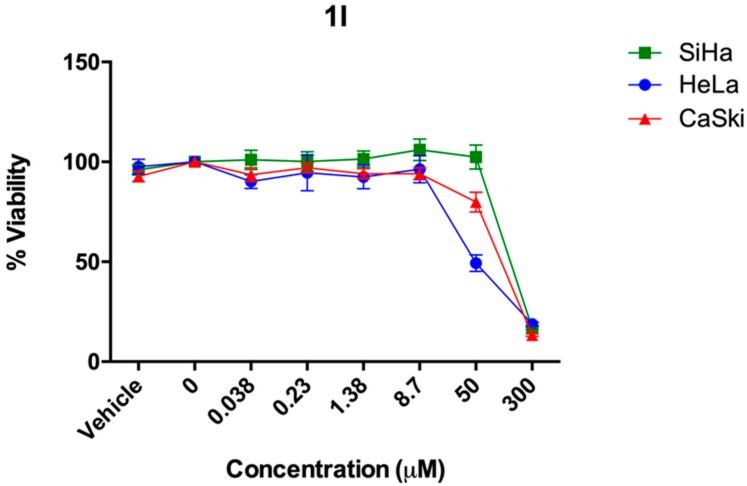
Cell viability of the compound **1l** against SiHa, HeLa, and CaSki.

It is worth highlighting the case of **1l**, which, at a concentration of 50 μM, impaired HeLa cells’ viability (49.3 ± 4.12%) (Figure 4). CaSki cells were also affected, but to a less extent, than HeLa at 79.9 ± 14.94%. At a concentration of 300 μM, all the cell lines were markedly affected with % viability of 16.8 ± 0.67%, 18.8 ± 1.03%, and 13.3 ± 0.66%, for SiHa, HeLa, and CaSki, respectively. Interestingly, at the highest concentration of 300 μM, CaSki was more sensitive than HeLa, which is the opposite to the pattern observed at 50 μM.

**Figure 5:**
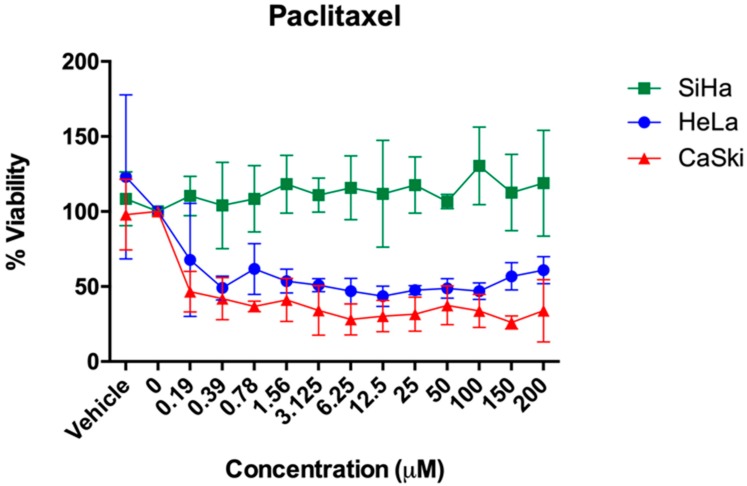
Cell viability of the reference drug Paclitaxel against HeLa, SiHa, and CaSki.

Paclitaxel (Figure 1) is widely known to specifically interact with tubulins and is one of the first line treatments for ovarian and breast cancer [40,41]. In this context, we used Paclitaxel as a standard and found that it affected HeLa and CaSki at a concentration of 0.19 μM at 48 h of exposure. SiHa showed a tendency to be more resistant (Figure 5).

A SAR analysis shows that compound **1h** (50.58 ± 4.33; 44.04 ± 14.45) was less cytotoxic than **1k** (22.7 ± 2.6; 24.9 ± 3.38) and **1l** (16.8 ± 0.67; 18.8 ± 1.03) against SiHa and HeLa, respectively; while it was considerably more active against CasKi (4.57 ± 4.54) in comparison to **1k** (16.2 ± 1.7) and **1l** (13.3 ± 0.66). The main structural difference between **1h** and **1k** or **1l** is that **1h** has a Falipamil’s pharmacophoric fragment, which interacts in different manners with the studied cell lines (Table 1). In the same context, **1l** (16.8 ± 0.67; 18.8 ± 1.03; 13.3 ± 0.66) was slightly more active than **1k** (22.7 ± 2.6; 24.9 ± 3.38; 16.2 ± 1.7), against SiHa, HeLa, and CaSki, despite their structural similarity. As it can be seen in Figure 1, between **1k** and **1l**, there is a chlorine (**1k**) instead hydrogen (**1l**) in their phenyl ring coming from the aldehyde moiety, as well as a morpholine ring (**1k**) instead piperidine (**1l)** in the amine moiety coming from the isocyanide reagents, which also results in a variation of their cytotoxicity values (% viability) (Table 1).

### 2.3. In Silico Studies

#### 2.3.1. Docking Assays

**Figure 6:**
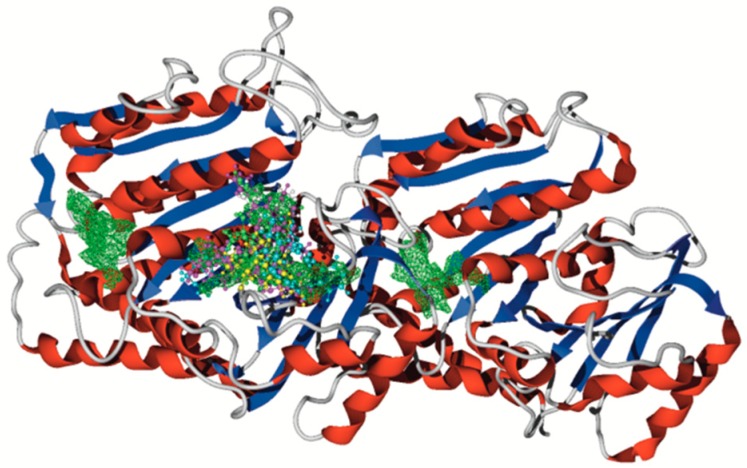
Molecular docking between the three active molecules **1h**, **k**–**l** (both enantiomers for each one) and the reference drug Paclitaxel, to the αβ-tubulin. Color key: The green surfaces are the cavities present in the protein.

The synthesis of polyheterocycles based on the pyrrole ring and their effect on the regulation of polymerization and depolymerization of tubulins have been reported, as well as the use of molecular docking for modelling their interactions with tubulins; just to cite a few [42,43,44,45,46]. Considering these results and since the compounds are pyrrole-based polyheterocycles, in silico studies were performed as a preliminary step to elucidate possible modes of action of the in vitro studied molecules. The results obtained through the molecular docking assays show that the RMSD was 0.75 Å, between one co-crystalized molecule in the selected biological target and the pose computed via the chosen MolDock [47] score function, corroborating a good precision in our method. The largest cavity in the αβ-tubulin is the site to perform the molecular docking of the three active molecules **1h, k**–**l** (both enantiomers for each one) and the reference drug Paclitaxel, because in the downloaded protein (PDB code: 1JFF) [48], the co-crystalized Paclitaxel is placed. Figure 6 shows the docking of the whole studied molecules, where the molecular coupling is clearly shown in the active cavity located in the center of the protein.

Considering the above-mentioned importance and influence of the tubulins to SiHa, HeLa, and CaSki cells, which have been reported in the context of cervical cancer [16,17,18] and analyzing the computed poses of all studied molecules in the largest (active) cavity, we can understand the reasons behind the cytotoxicity values for the three synthesized molecules **1h**, **k**–**l** in racemic form, at 300 μM against SiHa, HeLa, and CaSki cells, and the higher activity of Paclitaxel (<50% viability) from low concentrations (0.39 μM) against HeLa and CaSki (see Figure 5).

**Figure 7:**
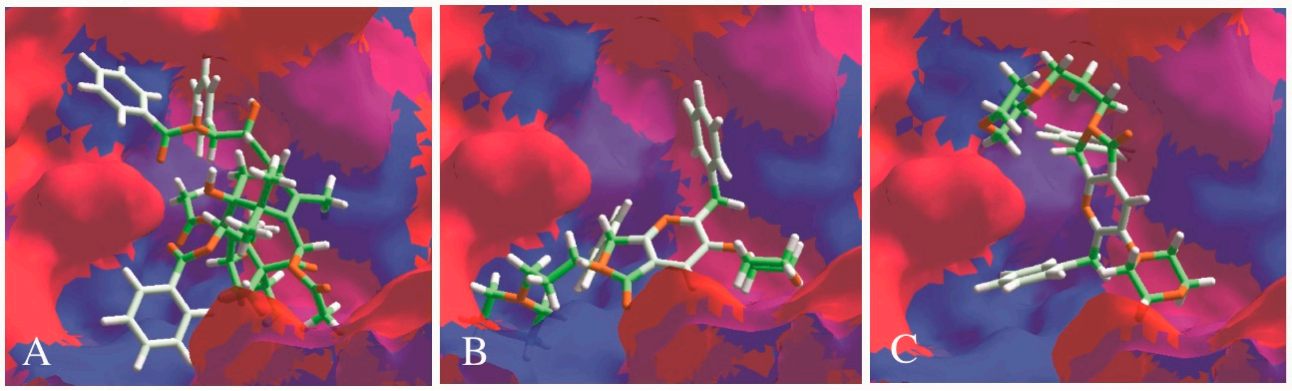
Hydrophobicity interactions between the αβ-tubulin with (**A**) Paclitaxel, (**B**) **1k** (enantiomer R), and (**C**) **1k** (enantiomer S). Color key: The blue color depicts hydrophobic surfaces, the red color depicts hydrophilic surfaces, green sticks depict hydrophobic moieties, and orange sticks depict hydrophilic moieties.

Moreover, Figure 7 shows the hydrophobicity interactions between the αβ-tubulin and the Paclitaxel (Figure 7A), and with the ligand **1k** (Figure 7B,C), considering both enantiomers, R and S. Analyzing the hydrophobic interaction of Paclitaxel, note a higher expansion into the cavity than **1k** (enantiomer R), showing the last interactions between its two benzene rings and the protein surface, while the Paclitaxel interacts with three rings. Since white color represents a neutral hydrophobic behavior, the hydrophobic character of benzene rings is well known. For this reason, the interaction of two benzenes with hydrophilic surfaces (Figure 7B) can promote less affinity between the biological target and the ligand, than the notorious interactions of the benzene moieties of Paclitaxel (Figure 7A), which interact with blue surfaces (hydrophobic surfaces). On the other hand, with the **1k** (enantiomer S), a better interaction can be promoted between the selected target and this ligand.

**Figure 8:**
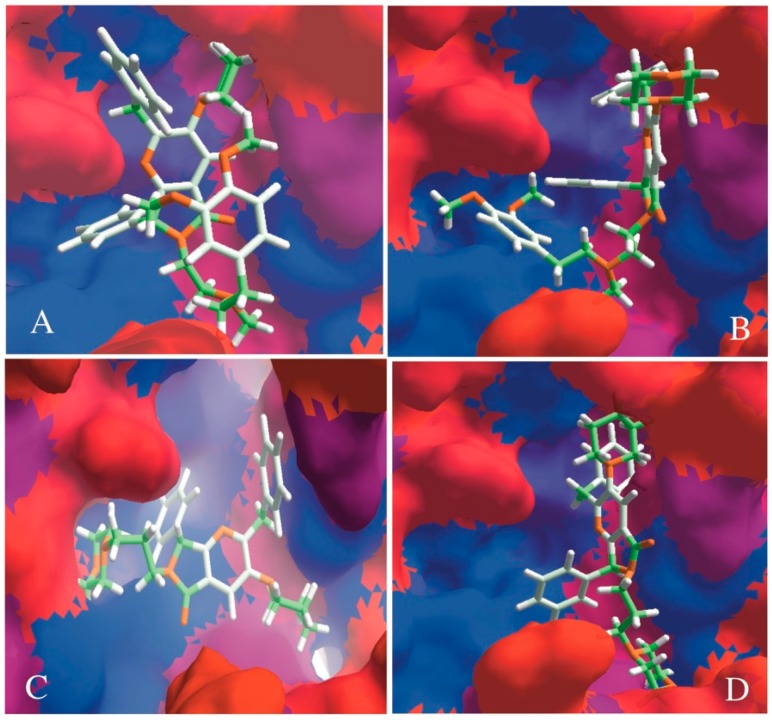
Hydrophobicity interactions between the αβ-tubulin with (**A**) **1l** (enantiomer R), (**B**) **1l** (enantiomer S), (**C**) **1h** (enantiomer R), and (**D**) **1h** (enantiomer S). Color key: The blue color depicts hydrophobic surfaces, the red color depicts hydrophilic surfaces, green sticks depict hydrophobic moieties, and orange sticks depict hydrophilic moieties.

As follows, Figure 8 shows hydrophobic interactions between the αβ-tubulin and the **1h** and **1l** ligands, considering both enantiomers. Note that **1l** (enantiomer R) (Figure 8A), which is a better ligand than **1h** and **1k** (both in racemic form) against two cellular lines (SiHa and HeLa), interacts with the three benzene rings, like Paclitaxel. However, the interaction sides are different and do not cover the posterior side as the Paclitaxel does effectively. Besides, Figure 8C shows the hydrophobic interactions between **1h** (enantiomer R) and the αβ-tubulin surface, which is similar to those for the **1k** interactions, but the chloride moiety of **1k** hinders stronger interactions. Additionally, the enantiomer S of polyheterocycle **1l** interacts with less sites than its enantiomer R, which perhaps means that the enantiomer R may exhibit better activity if it were in vitro studied in enantiomerically pure form. Moreover, the compound **1h** (enantiomer S) interacts orthogonally with the αβ-tubulin, with respect to its enantiomer R, resulting in a similar behavior to **1l**.

#### 2.3.2. QSAR and Pharmacophoric Modeling

Using the dragon software package [49,50], 4000 descriptors have been obtained for the studied molecules, which were screened with the mobydigs software [51]. Thus, a correlation was found between the half-maximal effective concentration ED_50_ (related to cell viability) for each cellular line and some structural and charge parameters, and with the compounds partition coefficient (see the Equations (1)–(3)).

ED_50_^SiHa^ = 261.555 − 35.810(nCrs) Q^2^_loop_ = 95.99; R^2^ = 0.991 (1)

ED_50_^HeLa^ = 102.076(nROR) − 18.141(nCbH) + 173.613 Q^2^_loop_ = 97.88; R^2^ = 0.996 (2)

ED_50_^CaSki^ = 82.161 (nBO) − 75.891(SCBO) + 795.812 Q^2^_loop_ = 71.41; R^2^ = 0.983 (3)

Notably, the coefficient of determination (R^2^) and the square correlation coefficient for leave-one-out cross-validation (Q^2^_loop_) for all the computed models are higher than 0.98 and 71, respectively, which means that the models are reliable. Equation (2) shows that the number of ethers (aliphatic, nROR) and the number of unsubstituted benzenes (*sp*^2^, nCbH) are the descriptors that present a better relationship with the ED_50_ against HeLa. At the same time, the ED_50_ against SiHa presents a relationship with the number of ring secondary C (*sp*^3^, nCrs). Moreover, the number of non-H bonds (nBO) and the sum of conventional bond orders (SCBO) are the descriptors presenting the best correlation with the ED_50_ against CaSki. Thus, by analyzing the selected descriptors (as the number of ethers, unsubstituted benzenes, secondary rings, and non-H bonds) and considering the results obtained via the docking assays, it can be proposed that the number of unsubstituted benzenes and secondary rings are the most important descriptors for the present analysis, due to the hydrophobic interactions that reveals the importance of benzenes in the ligand–target interactions.

Based on the QSAR shown above, the high importance of benzenes for further anticancer drug design, focused on a cervical neoplastic process, and considering the αβ-tubulin as the biological target, is obvious. Above, due to the fact that the determinant coefficient is higher than 0.98 in all the cases and the Q^2^_loop_ is major to 60, validated data were obtained in all cases.

**Figure 9:**
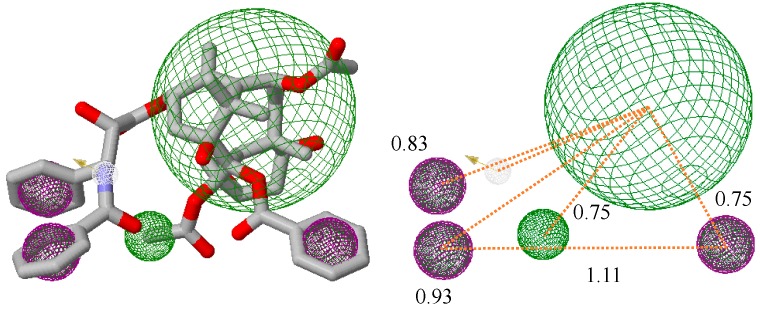
Pharmacophoric model. Green spheres represent a hydrophobic segment, purple spheres an aromatic segment, and the gray sphere a hydrogen donor segment. Distance units = Å.

Through the ZINCPharmer server [52], the pharmacophoric model has been computed, based on the bioactive structure of the Paclitaxel in the αβ-tubulin dimer, which shows a higher hydrophobic segment, about 4 Å of diameter on the right site (depicted in green color, Figure 9). Moreover, the pharmacophoric model shows another hydrophobic segment, only 0.75 Å behind the biggest hydrophobic site, and a hydrogen donor segment about the same distance, but on the left site of the green sphere. Finally, three aromatic-hydrophobic segments are distributed surrounding the higher hydrophobic zone, at 0.75, 0.83, and 0.93 Å distance, which are responsible for the stronger interactions between the biological target and the control drug Paclitaxel. It is noteworthy that the hydrophobic behavior in a molecule is the most important factor in this study.

## 3. Experimental Part

### 3.1. Synthesis

#### 3.1.1. General Information, Instrumentation, Software, and Chemicals

^1^H and ^13^C nuclear magnetic resonance (NMR) spectra were acquired on a Bruker AMX Advance III spectrometer (500 MHz, Fällande, Uster, Switzerland). The solvents used for NMR experiments were deuterated chloroform (CDCl_3_) or deuterated methanol (CD_3_OD). Chemical shifts are reported in parts per million (δδδδδ/ppm). Coupling constants are reported in Hertz (*J*/Hz). Internal reference for NMR spectra was tetramethylsilane (TMS) at 0.00 ppm. Multiplicities of the signals are reported using the standard abbreviations: singlet (s), doublet (d), triplet (t), quartet (q) and multiplet (m). NMR spectra were analyzed using the MestReNova software (Ver. 12.0.0-20080). Infrared (IR) spectra were acquired on a Perkin Elmer 1600 spectrometer (Norwalk, CT, USA) using the attenuated total reflectance (ATR) method. The maximum absorbance peaks are reported in reciprocal centimeters (υ_max_/cm^−1^). IR spectra were analyzed using the Report Builder software (Ver. 2.01). High-resolution mass spectroscopy (HRMS) spectra were acquired by Electrospray ionization (ESI) on a Micro-TOF II spectrometer Bruker Daltonics GmbH (Bremen, Germany). HRMS samples were injected directly (Apollo source) and analyzed by time of flight (TOF). HRMS spectra were analyzed using the Compass 1.5 analysis software. Melting points were determined on a Fisher–Johns apparatus (Suwanee, GA, USA) and are uncorrected. Microwave-assisted reactions were performed in closed-vessel mode on a CEM Discover MW-reactor (Matthews, North Carolina, CA, USA). Reaction progress was monitored by thin-layer chromatography (TLC) and the spots were visualized under ultraviolet (UV) light (254 or 365 nm). Flash columns packed with silica-gel 60 and glass preparative plates (20 × 20 cm) coated with silica-gel 60 doped with UV indicator (F254) were used to purify the products. Mixtures of hexanes (Hex) and ethyl acetate (EtOAc) in 1:1 (*v*/*v*) proportion were used to run TLC, silica-gel columns, preparative plates, and to measure the retention factor (R*_f_*) values (using the same mobile phase for all the experiments). All starting reagents and solvents were used as received (without further purification, distillation, nor dehydration). Chemical structures were drawn using the ChemDraw Professional software (Ver. 15.0.0.106, Perkin Elmer Informatics, Cambridge, MA, USA). The purity for all synthesized products (>99%) was assessed by NMR.

#### 3.1.2. Synthesis and Characterization of the Pyrrolo[3,4-b]pyridin-5-ones **1k**–**l**

##### General Procedure (GP)

The corresponding aldehydes **2a**–**b** (1.0 equiv.) and the amine **3** (0.1 mmol, 1.0 equiv.) were placed in a sealed CEM Discover microwave reaction tube (10 mL) and diluted in benzene [1.0 mL]. Then, the mixture was stirred and heated using microwave irradiation (65 °C, 100 W) for 5 min, and scandium (III) triflate (0.03 equiv.) was added. The mixture was stirred and heated using microwave irradiation (65 °C, 100 W) for 5 min, and then the corresponding isocyanides **4a**–**b** (1.2 equiv.) were added. The new mixture was stirred and again heated using microwave irradiation (80 °C, 100 W) for 15 min, and then maleic anhydride **5** (1.4 equiv.) was added. Finally, the reaction mixture was stirred and heated using microwave irradiation (80 °C, 100 W) for 15 min. Then, the solvent was removed to dryness under vacuum. The crude was extracted using dichloromethane (3 × 25.0 mL) and Na_2_CO_3_ aq. (3 × 25 mL), and then washed with brine (3 × 25 mL). The organic layer was dried using anhydrous Na_2_SO_4_, filtered, and concentrated to dryness under vacuum. The new crude was purified by silica-gel column chromatography followed by preparative TLC using mixtures of hexanes (Hex) and ethyl acetate (EtOAc) in 1:1 (*v*/*v*) proportion as mobile phase to isolate the corresponding pyrrolo[3,4-*b*]pyridin-5-ones **1k**–**l** as a racemic mixtures.

##### 2-Benzyl-7-(4-chlorophenyl)-3-morpholino-6-(3-morpholinopropyl)-6,7-dihydro-5H-pyrrolo [3,4-b]pyridin-5-one (**1k**)

According to the GP, 4-chlorobenzaldehyde (0.3 mmol), 3-morpholinopropan-1-amine (0.3 mmol), scandium (III) triflate (0.009 mmol), 2-isocyano-1-morpholino-3-phenylpropan-1-one (0.36 mmol), and maleic anhydride (0.42 mmol) were reacted together in PhH (1.0 mL) to afford **1k** (73.4 mg, 0.135 mmol, 46%) as a white oil; R*_f_* = 0.06 (Hex–AcOEt = 1:1, *v*/*v*); FT-IR (ATR) ν_max_/cm^−1^ 1760; ^1^H NMR (500 MHz, CDCl_3_): δ 1.83–1.89 (m, 2H), 2.49–2.60 (m, 6H), 2.81–2.84 (m, 4H), 2.98–3.05 (m, 1H), 3.72–3.75 (m, 4H), 3.79–3.82 (m, 4H), 3.93–3.99 (m, 1H), 4.20 (d, *J* = 13.9 Hz, 1H), 4.29 (d, *J* = 13.9 Hz, 1H), 5.50 (s, 1H), 7.12 (d, *J* = 8.4 Hz, 2H), 7.15–7.22 (m, 5H), 7.34 (d, *J* = 8.5 Hz, 2H), 7.84 (s, 1H); ^13^C{^1^H} NMR (126 MHz, CD_3_OD): δ 23.8, 38.4, 39.3, 52.7, 52.8, 55.4, 64.9, 65.5, 66.7, 123.8, 124.0, 125.8, 127.9, 128.4, 128.9, 129.6, 134.2, 134.5, 139.2, 148.5, 160.2, 162.5, 167.8; HRMS (ESI^+^): *m*/*z* calcd for C_31_H_36_ClN_4_O_3_ [M + H]^+^ 547.2476, found 547.2450.

##### 2-Benzyl-6-(3-morpholinopropyl)-7-phenyl-3-(piperidin-1-yl)-6,7-dihydro-5*H*-pyrrolo[3,4-*b*] pyridin-5-one (**1l**)

According to the GP, benzaldehyde (0.3 mmol), 3-morpholinopropan-1-amine (0.3 mmol), scandium (III) triflate (0.009 mmol), 2-benzyl-3-oxo-3-(piperidin-1-yl)propanenitrile (0.36 mmol), and maleic anhydride (0.42 mmol) were reacted together in PhH (1.0 mL) to afford **1l** (69.1 mg, 0.135 mmol, 45%) as a white oil; R*_f_* = 0.05 (Hex–AcOEt = 1:1, *v*/*v*); FT-IR (ATR) ν_max_/cm^−1^ 1759; ^1^H NMR (500 MHz, CDCl_3_): δ 1.57–1.63 (m, 2H), 1.69–1.75 (m, 4H), 1.76–1.85 (m, 2H), 2.38–2.48 (m, 6H), 2.76–2.83 (m, 4H), 3.01–3.08 (m, 1H), 3.66–3.70 (m, 4H), 3.93–3.99 (m, 1H), 4.20 (d, *J* = 13.7 Hz, 1H), 4.28 (d, *J* = 13.7 Hz, 1H), 5.49 (s, 1H), 7.11–7.23 (m, 7H), 7.35–7.39 (m, 3H), 7.83 (s, 1H); ^13^C{^1^H} NMR (126 MHz, CDCl_3_): δ 23.9, 24.8, 26.4, 38.6, 39.8, 53.3, 54.3, 56.0, 65.6, 66.5, 123.1, 123.8, 125.9, 128.0 (2), 128.6, 128.8, 128.9, 135.8, 139.5, 149.4, 159.5, 161.9, 167.6; HRMS (ESI^+^): *m*/*z* calcd for C_32_H_39_N_4_O_2_ [M + H]^+^ 511.3073, found 511.3048.

### 3.2. In Vitro Studies

SiHa, HeLa, and CaSki cells were cultured in DMEM advanced (Gibco™), supplemented with 8% fetal bovine serum (FBS Gibco™), 2x glutamine (Gibco™), and 1x penicillin and streptomycin (supplied by Gibco™), and cultured at 37 °C and 5% CO_2_. For viability in vitro studies, cells were trypsinized (Gibco™) and distributed in 96-well plates in a density of 50,000 cells per well in DMEM-advanced medium supplemented with 8% of FBS and 2x glutamine as previously described [53].

The compounds (in racemic form) were tested using the following concentrations: 300, 50, 8.3, 1.38, 0.23, and 0.038 μM for 48 h. For Paclitaxel experiments, the following concentrations were tested: 200, 150, 100, 50, 25, 12.5, 6.25, 3.125, 1.56, 0.78, 0.39, and 0.19 μM for 48 h. DMSO was used to prepared stock solutions of the compounds and its final concentration was less than 0.2% in the final dilutions. Viability was determined by MTS cell proliferation assay following the manufacturer instructions for CellTiter 96 Assay (Promega). The compounds were tested in duplicates and repeated 4 times (*n* = 8) and the Paclitaxel experiments were performed in duplicates two times (*n* = 4). Experimental data were analyzed and graphed using Graphpad Prism. ED_50_ values were generated using the AAT Bioquest calculator [54]

### 3.3. In Silico Studies

The ligands have been modeled and optimized with the Gaussian 09 package [55], using the universal force field (UFF) [56]. Moreover, the biological target selected has been downloaded from the protein data bank (PDB), with eh PDB code 1JFF [48] and adjusted to pH 7, deleting solvent and correcting resides with the software Chimera [57]. However, the molecular docking was performed via the scoring function MolDock [58] with the Molegro virtual docker package (MVD) [47]. Also, the molecular descriptors were computed with the dragon software [49,50] and the QSAR models have been obtained with the mobydigs package [51]. Finally, we obtained the pharmacophoric model using the ZINCPharmer server [52].

## 4. Conclusions

A series of 12 pyrrolo[3,4-*b*]pyridin-5-ones (10 already reported and 2 new) in racemic form were studied in vitro against SiHa, HeLa, and CaSki cancer cervical human cell lines, obtaining moderate viability values. Through the in silico assays, it can be concluded that pyrrolo[3,4-*b*]pyridin-5-ones need some hydrophobic sites to bind better with the αβ-tubulin, in counterpart of Paclitaxel, which presents more hydrophobic-aromatic sites and can be coupled better with the biological target, due to the hydrophobic interactions. Furthermore, these hydrophobic-aromatic moieties play a key role in the ligand–target complex. The QSAR study revealed that the six members rings are the most significant fragments, being present in all computed models for the cell cultures, SiHa, Hela, and CaSki.

## Figures and Tables

**Table 1:** Effect of the studied compounds **1h**, **1k**, and **1l** on viability of cervical cancer cell lines SiHa, HeLa, and CaSki.

% Cell Viability ± SD (ED_50_ Values)			
Compound	SiHa	HeLa	CaSki
**1h**[a]	50.58 ± 4.33 (281.09)	44.04 ± 14.45 (180.66)	4.57 ± 4.54 (288.76)
**1k**[a]	22.7 ± 2.6 (212.92)	24.9 ± 3.38 (206.02)	16.2 ± 1.7 (237.04)
**1l**[a]	16.8 ± 0.67 (212.64)	18.8 ± 1.03 (64.44)	13.3 ± 0.66 (119.27)
[a] *n* = 8 at 300 μM			

## Supplementary Materials

The following are available online, Structures of all compounds tested against SiHa, HeLa, and CaSki cell lines. Plots of initial viability assays. Copies of ^1^H and ^13^C NMR spectra of the new products **1k**–**l**. Docking data.
