# Supplementary material for: Synthesis of Pyrrolo[3,4-b]pyridin-5-ones via Multicomponent Reactions and In Vitro–In Silico Studies Against SiHa, HeLa, and CaSki Human Cervical Carcinoma Cell Lines
Source: Molecules. 2019 Jul 22;24(14):2648. doi: 10.3390/molecules24142648 (PMC6680468; doi:10.3390/molecules24142648)
Supplement: Supplementary file 1 [file molecules-24-02648-s001.pdf]

S U P P L E M E N T A R Y   M A T E R I A L

Synthesis of pyrrolo[3,4-*b*]pyridin-5-ones via multicomponent reactions and *in vitro–in silico* studies against SiHa, HeLa and CaSki human cervical carcinoma cell lines

Daniel Segura-Olvera [1], Ailyn N. García-González [1], Ivette Morales-Salazar [1], Alejandro Islas-Jácome [1], Yareli Rojas-Aguirre [2], Ilich A. Ibarra [3], Erik Díaz-Cervantes [4,*], Sofía Lizeth Alcaraz-Estrada [5,*], and Eduardo González-Zamora [1,*]

[1] Departamento de Química, Universidad Autónoma Metropolitana-Iztapalapa, San Rafael Atlixco 186, Col. Vicentina, C.P. 09340, Iztapalapa, Ciudad de México; mudo-2@hotmail.com (D.S.-O.); natgg@outlook.com (A.N.G.-G.); ivette649_tatu@hotmail.com (I.M.-S.); aij@xanum.uam.mx (A.I.-J.)

[2] Departamento de Polímeros, Instituto de Investigaciones en Materiales, Universidad Nacional Autónoma de México, Circuito Exterior S/N, Ciudad Universitaria, C.P. 04510, Coyoacán, Ciudad de México; yareli.rojas@materiales.unam.mx (Y.R.-A.)

[3] Laboratorio de Fisicoquímica y Reactividad de Superficies, Instituto de Investigaciones en Materiales, Universidad Nacional Autónoma de México, Circuito Exterior S/N, Ciudad Universitaria, C.P. 04510, Coyoacán, Ciudad de México; argel@unam.mx (I.A.I.)

[4] Departamento de Alimentos, Centro Interdisciplinario del Noreste, Universidad de Guanajuato, C.P. 37975, Tierra Blanca, Guanajuato, México.

[5] Departamento de Biología Molecular, División de Medicina Genómica, Centro Médico Nacional 20 de Noviembre, ISSSTE, Félix Cuevas 540, Col. Del Valle Sur, C.P. 03100, Benito Juárez, Ciudad de México.

* Correspondence: e.diaz@ugto.mx (E.D.-C.); sofializeth@gmail.com (S.L.A.-E.); egz@xanum.uam.mx (E.G.-Z.)

C O N T E N T S

1. **Compounds initially tested**	S2–S4
2. **Initial viability tests**	S4–S5
3. **NMR spectra of the new products**	S6–S7
4. **Docking**	S8–S22
5. **References**	S22

1. Compounds initially tested

Table S1. Compounds and Origin

Compound	Origin
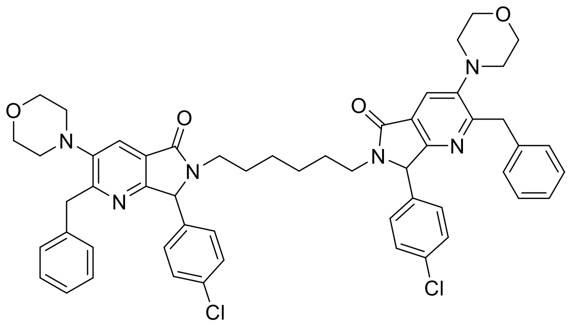 **1a**	[Ref. S1]
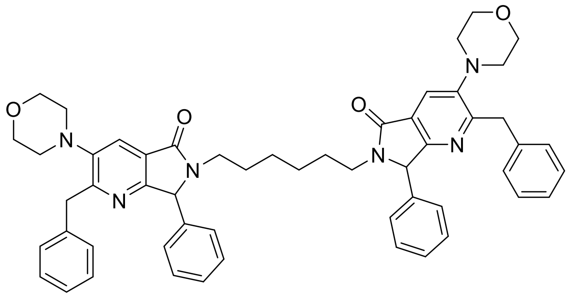 **1b**	[Ref. S1]
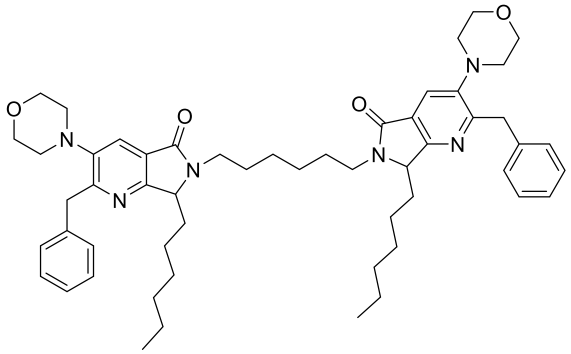 **1c**	[Ref. S1]
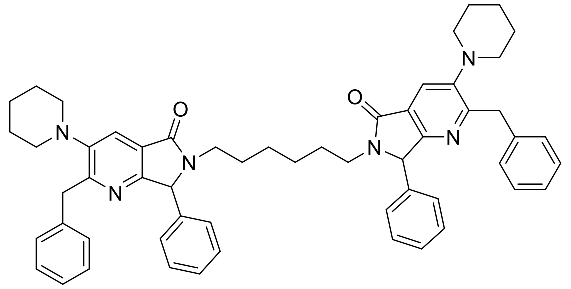 **1d**	[Ref. S1]

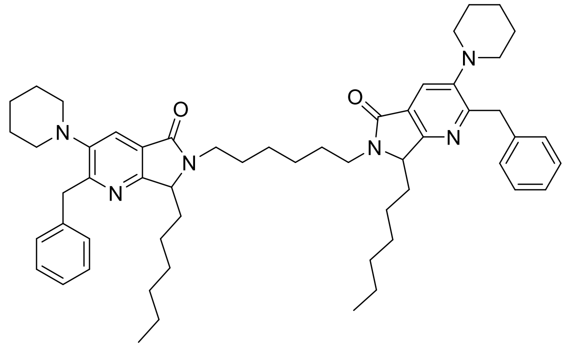<br>**1e**	[Ref. S1]
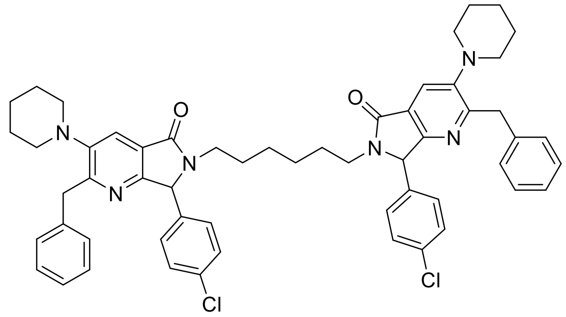<br>**1f**	[Ref. S1]
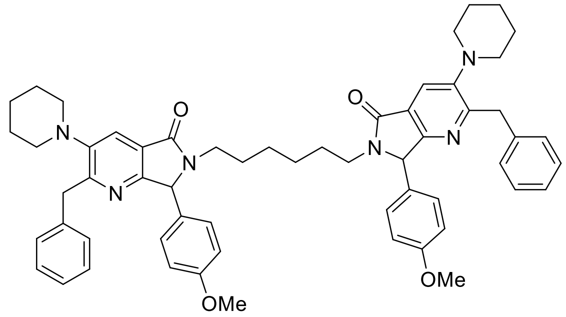<br>**1g**	[Ref. S1]
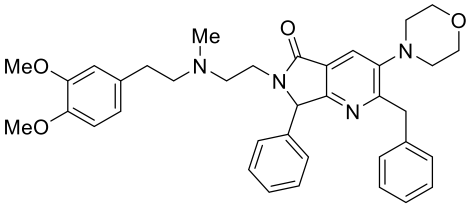<br>**1h**	[Ref. S2]
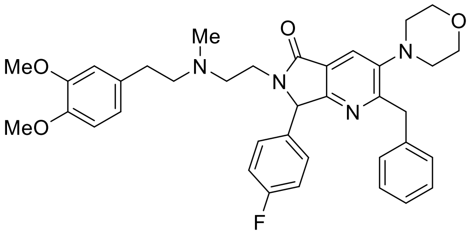<br>**1i**	[Ref. S2]

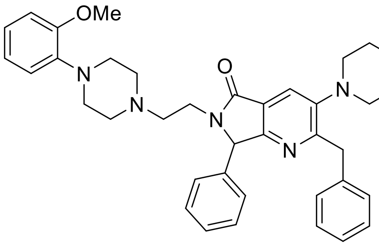 **1j**	[Ref. S2]
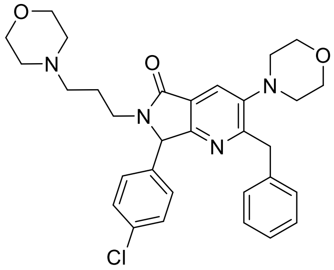 **1k**	New product
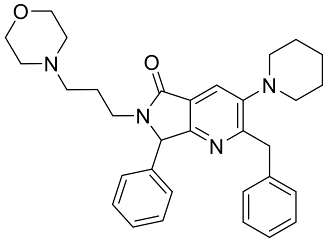 **1l**	New product

2. Initial viability tests

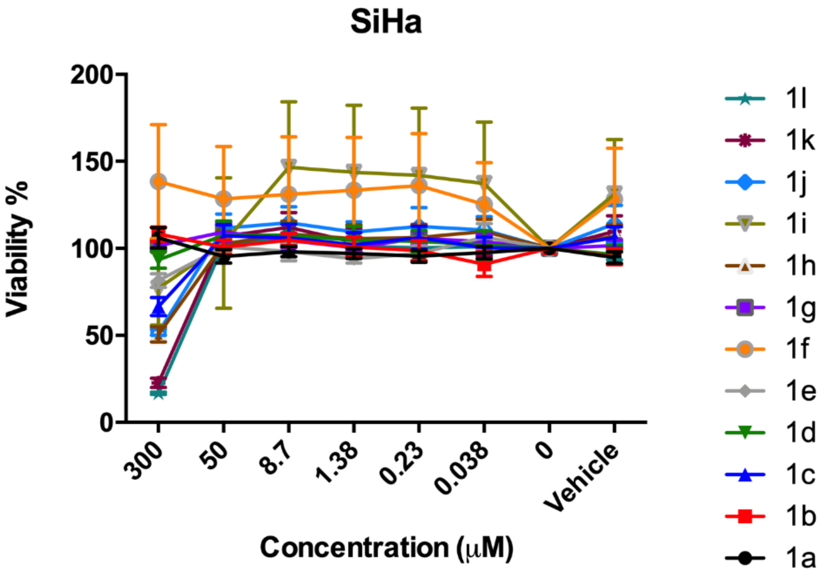

Figure S1. All compounds initially tested against SiHa cell line

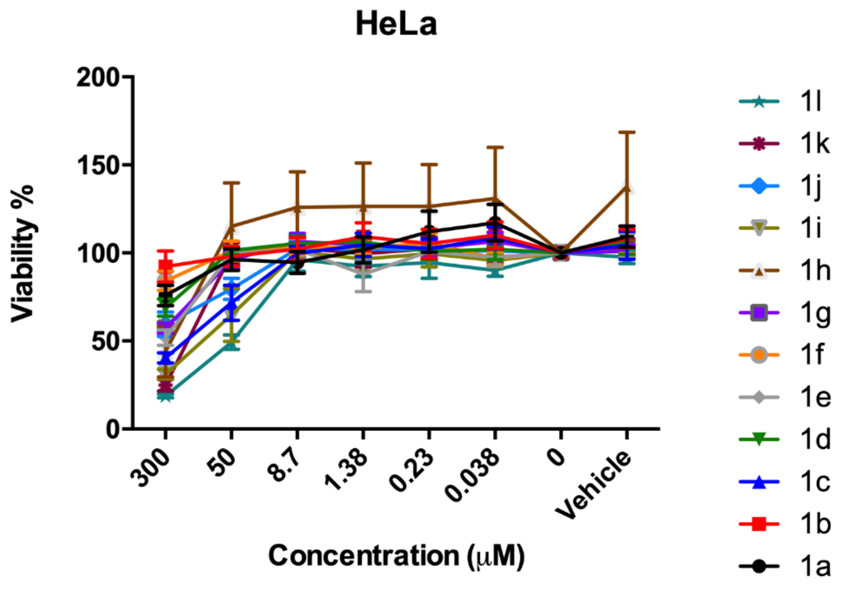

Figure S2. All compounds initially tested against HeLa cell line

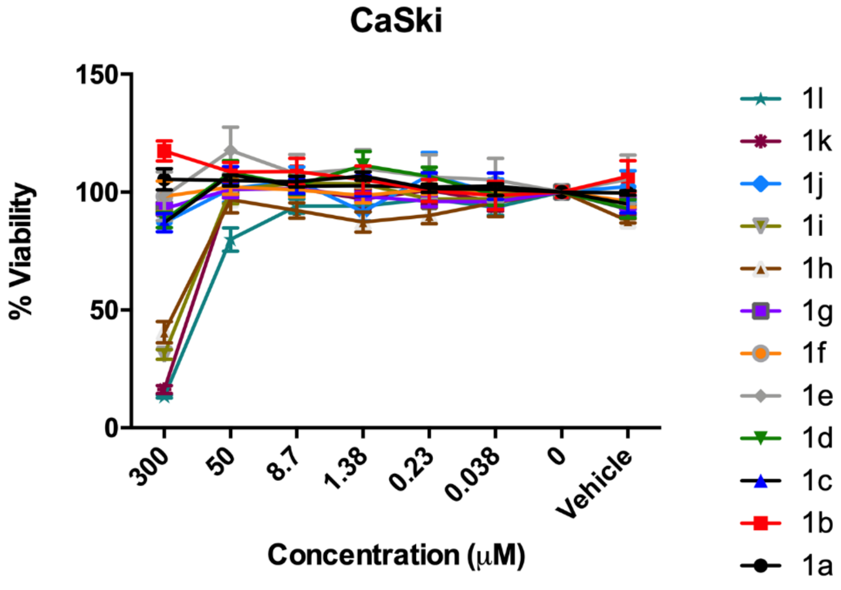

Figure S3. All compounds initially tested against CaSki cell line

The evaluation of the twelve compounds in SiHa, HeLa and CaSki cells was carried out as described in section 3.2 of the manuscript. Compounds **1h**, **1k** and **1l** had an effect in cell viability in each of these cell lines.

3. **Spectra of the new compounds 1k–l**

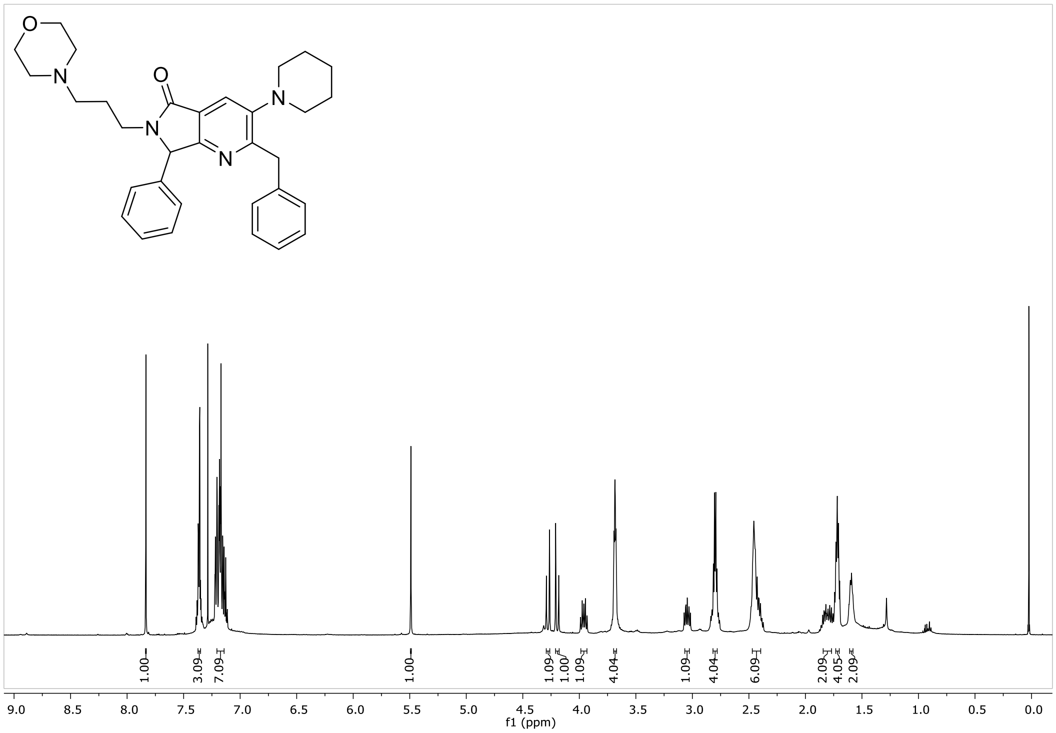

Figure S4. ^{1}H NMR spectrum of the compound **1k**

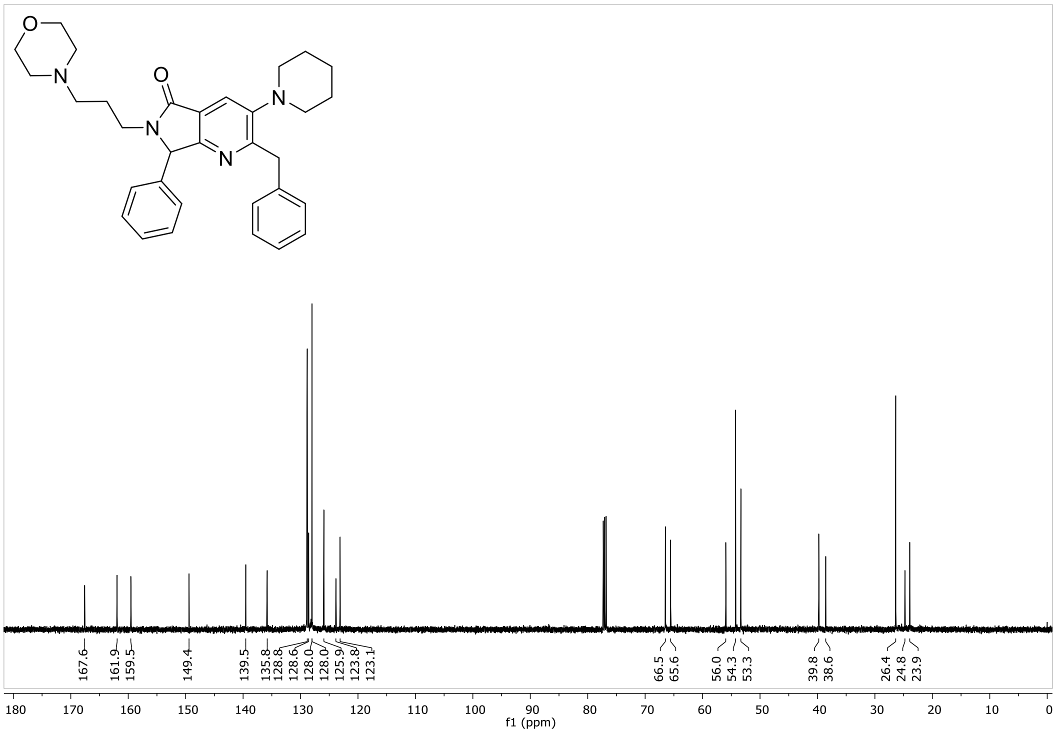

Figure S5. ^{13}C NMR spectrum of the compound **1k**

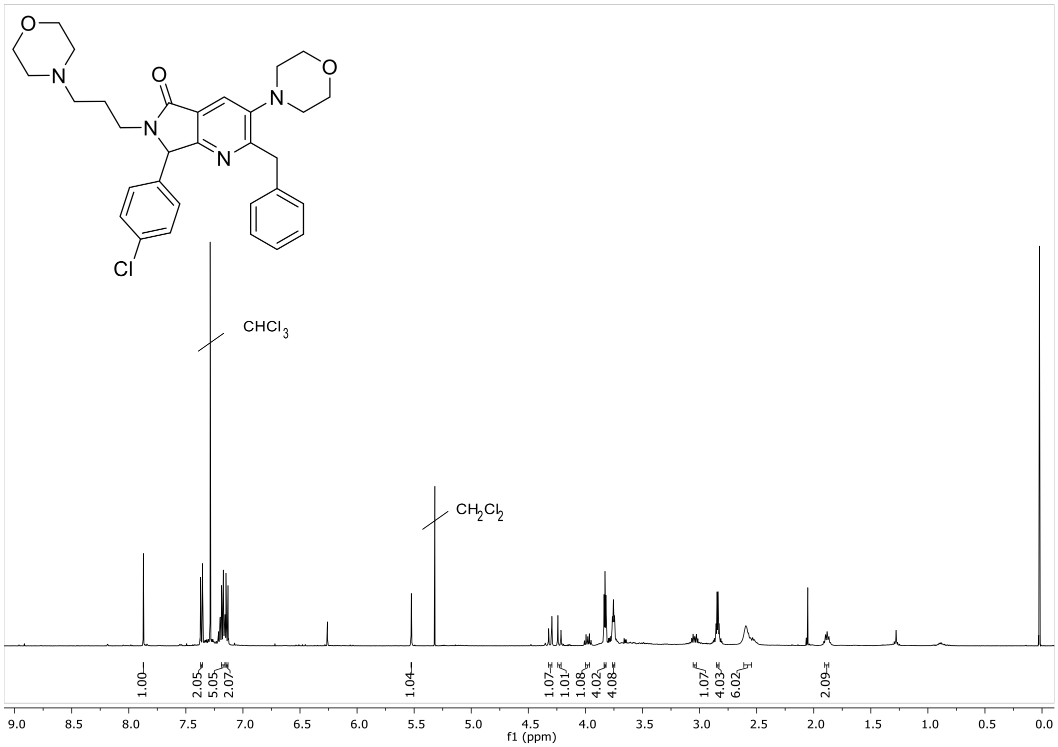

Figure S6. ^{1}H NMR spectrum of the compound **1l**

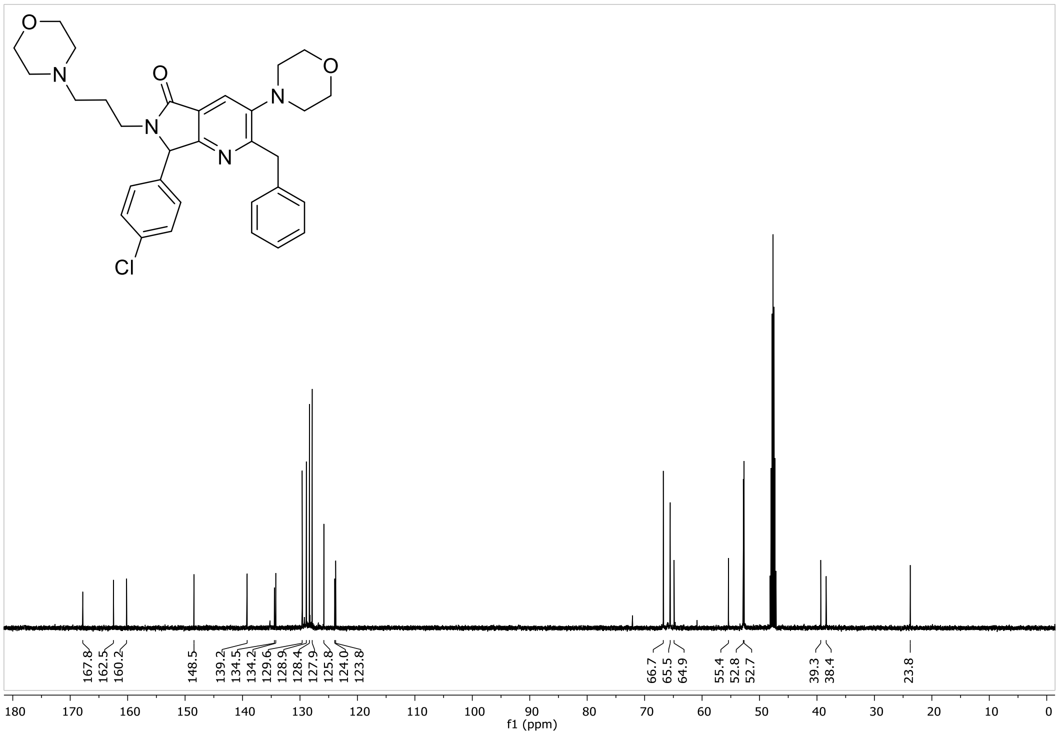

Figure S7. ^{13}C NMR spectrum of the compound **1l**

4. Docking

The chosen MOLDOCK score function was calibrated with its co-crystalized ligand (Figure S8), which demonstrate that our method is trustworthy with an RMSD of 0.750 Å.

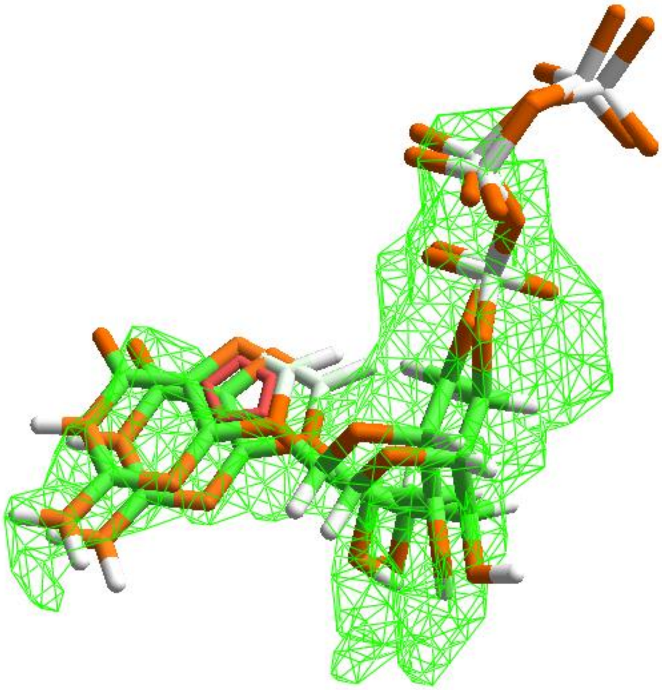

Figure S8. Co-crystalized ligand and computed pose.

Table S2. Moldock Scores (ΔG_b)

Ligand	Moldock Scores (Kcal/mol)
**1h** (enantiomer R)	-141.59
**1h** (enantiomer S)	-164.86
**1k** (enantiomer R)	-139.24
**1k** (enantiomer S)	-154.13
**1l** (enantiomer R)	-199.91
**1l** (enantiomer S)	-194.39
**Paclitaxel**	-175.37

Table S3. Bioactive conformation for the three molecules **1h (R and S)**, **1k (R and S)** and **1l (R and S)**

1h (enantiomer R)

C	2.5244000000	-17.8890000000	12.0920000000
C	-1.3475000000	-19.1185000000	11.1835000000
N	-2.0854000000	-17.8770000000	11.1285000000

C	-1.0603000000	-16.8429000000	11.4700000000
C	2.3807000000	-19.2951000000	11.9190000000
C	1.0984000000	-19.8173000000	11.6239000000
C	0.0411000000	-18.9236000000	11.5108000000
C	0.2198000000	-17.5908000000	11.6719000000
N	1.4361000000	-17.0704000000	11.9693000000
C	-3.1950000000	-17.9044000000	12.1023000000
H	0.9320000000	-20.8766000000	11.4813000000
C	3.8586000000	-17.2690000000	12.4099000000
O	-1.8383000000	-20.2023000000	10.9054000000
H	4.4099000000	-22.8242000000	13.9993000000
H	-1.2891000000	-16.3168000000	12.4254000000
H	-2.5086000000	-16.7245000000	9.1314000000
H	-4.1387000000	-18.1928000000	11.5861000000
C	-3.4069000000	-16.5112000000	12.7109000000
H	-3.0074000000	-18.6560000000	12.9037000000
H	-2.9787000000	-16.4956000000	13.7321000000
C	-4.9073000000	-16.1564000000	12.7632000000
H	-2.8606000000	-15.7756000000	12.0844000000
H	-5.0446000000	-15.2433000000	13.3836000000
H	-5.4537000000	-16.9724000000	13.2851000000
H	-6.4972000000	-14.2099000000	9.5293000000
N	-5.5345000000	-15.9355000000	11.4414000000
C	-6.6840000000	-16.8136000000	9.4692000000
C	-5.9158000000	-17.1757000000	10.7460000000
C	-4.7577000000	-15.0546000000	10.5532000000
C	-5.5743000000	-14.7833000000	9.2838000000
O	-5.8943000000	-15.9956000000	8.6245000000
H	-4.9686000000	-14.1563000000	8.5963000000
H	-7.6455000000	-16.3115000000	9.7219000000
H	-6.9324000000	-17.7486000000	8.9243000000
H	-5.0189000000	-17.7675000000	10.4607000000
H	-6.5683000000	-17.7955000000	11.3989000000
H	-3.7957000000	-15.5266000000	10.2584000000
H	-4.5469000000	-14.0882000000	11.0606000000
C	-0.9345000000	-15.8495000000	10.3384000000
C	0.0148000000	-14.8148000000	10.4085000000
C	0.1303000000	-13.8905000000	9.3658000000
C	-0.6989000000	-13.9898000000	8.2457000000
C	-1.6472000000	-15.0126000000	8.1667000000
C	-1.7680000000	-15.9382000000	9.2073000000
H	0.6651000000	-14.7210000000	11.2694000000
H	0.8633000000	-13.0965000000	9.4263000000
H	3.8460000000	-18.0031000000	17.0320000000
H	-2.2897000000	-15.0881000000	7.2991000000
N	3.5368000000	-20.1539000000	12.0468000000
C	5.2413000000	-21.5071000000	10.8849000000
C	3.9353000000	-20.7174000000	10.7422000000

C	3.3077000000	-21.2114000000	13.0481000000
C	4.5984000000	-22.0048000000	13.2728000000
C	5.0989000000	-22.5976000000	11.9516000000
H	5.3761000000	-21.3332000000	13.6987000000
H	6.0657000000	-20.8184000000	11.1679000000
H	5.5013000000	-21.9735000000	9.9104000000
H	3.1463000000	-21.3889000000	10.3357000000
H	4.1016000000	-19.8943000000	10.0119000000
H	2.4997000000	-21.9050000000	12.7230000000
H	3.0052000000	-20.7537000000	14.0161000000
H	4.0458000000	-16.4322000000	11.7022000000
H	4.6683000000	-18.0144000000	12.2529000000
H	3.7385000000	-18.7844000000	14.6949000000
C	3.9153000000	-16.8010000000	13.8410000000
C	4.0382000000	-15.4314000000	14.1340000000
C	4.1012000000	-14.9976000000	15.4614000000
C	4.0335000000	-15.9244000000	16.5051000000
C	3.8984000000	-17.2862000000	16.2229000000
C	3.8360000000	-17.7249000000	14.8968000000
H	4.0921000000	-14.7029000000	13.3342000000
H	4.2027000000	-13.9427000000	15.6811000000
H	4.0838000000	-15.5871000000	17.5322000000
H	-0.6073000000	-13.2735000000	7.4395000000
H	4.3845000000	-23.3731000000	11.5976000000
H	6.0833000000	-23.0864000000	12.1149000000

1h (enantiomer S)

C	2.07015510	1.34595940	0.21477952
C	-1.60639570	3.05378035	1.17115427
N	-2.38287423	1.92902571	1.64101910
C	-1.46725824	0.75057673	1.43298298
C	2.00442875	2.75396667	0.01287375
C	0.79336247	3.42745217	0.30198722
C	-0.27617719	2.67517065	0.77061683
C	-0.17293629	1.33660413	0.95697186
N	0.97599326	0.67077069	0.68029341
C	-3.71312256	1.88831473	0.99487398
H	0.68761487	4.49527843	0.16519083
C	3.31963577	0.56527590	-0.09507144
O	-2.00158631	4.20940249	1.21453731
H	3.96909140	5.13381294	-3.35960688
H	-2.28891166	-1.80293456	2.00580977
C	4.30563922	0.72816119	2.22517289
H	-4.17583281	2.90065063	1.01928425
C	-4.66386413	0.95778689	1.76290802

H	-3.64118338	1.62090394	-0.07693839
H	-4.14244389	0.02464414	2.03369591
C	-5.92612323	0.64627859	0.93135302
H	-4.95099163	1.46209494	2.71046242
H	-5.62958695	0.35245591	-0.10014515
H	-6.52390298	1.57811966	0.82870951
H	-8.13800483	-2.79619177	1.20680441
N	-6.78914532	-0.41328459	1.49857863
C	-8.21624725	-1.24441849	3.30402159
C	-7.23004253	-0.15043239	2.87840506
C	-6.21597567	-1.76529247	1.37815578
C	-7.24430161	-2.78997105	1.87162510
O	-7.61480031	-2.52357059	3.21254287
H	-6.79041465	-3.80234518	1.82471738
H	-9.14249725	-1.19596077	2.68719224
H	-8.51226864	-1.06775190	4.35955493
H	-6.36851730	-0.15897864	3.58205377
H	-7.73617839	0.83789847	2.93518976
H	-5.29302431	-1.86959534	1.98754149
H	-5.97115331	-1.98510999	0.31597477
C	5.34451933	1.07426877	3.09441904
C	6.52923006	1.61624177	2.58920495
C	6.67863194	1.80909276	1.21343423
C	5.64183163	1.46578480	0.34039201
H	3.39275614	0.30826977	2.62929809
H	5.23112094	0.92200196	4.15994332
H	7.33213118	1.88474651	3.26345542
H	5.73639220	6.05874005	-1.79698212
H	7.59678718	2.22863528	0.82311764
H	4.06835614	6.57456050	-1.36312229
N	3.16282618	3.46056804	-0.48639006
C	4.93816490	5.10807284	-0.01506593
C	3.63225790	4.47444593	0.47797159
C	2.89661569	4.05207091	-1.81023935
C	4.18368465	4.65645411	-2.37935962
C	4.75564624	5.70151455	-1.41582401
H	4.93160574	3.84911981	-2.54101143
H	5.73999186	4.33982353	-0.04094309
H	5.25110392	5.90890579	0.68881820
H	2.87208976	5.27427109	0.62349835
H	3.82584495	3.99497663	1.46328644
H	2.11398337	4.84157318	-1.74815752
H	2.54108917	3.26349153	-2.51012892
H	3.60248765	0.74551613	-1.15510095

H	3.11937023	-0.52353989	0.00654844
H	5.77092019	1.62830598	-0.72249806
C	4.44252616	0.93104394	0.84091709
H	-1.27864040	0.29234434	2.42810373
C	-1.94535679	-0.32445334	0.45788910
C	-1.96664865	-0.10078111	-0.93474986
C	-2.39830976	-1.10341921	-1.80752946
C	-2.79540842	-2.34487762	-1.30670945
C	-2.75027267	-2.59015780	0.06705482
C	-2.31666665	-1.59189199	0.94409610
H	-1.66042460	0.85452053	-1.34252893
H	-2.42423167	-0.91752173	-2.87341747
H	-3.13211042	-3.11914795	-1.98380980
H	-3.05211092	-3.55495719	0.45367670

1k (enantiomer R)

C	2.6101000000	-18.0498000000	12.2326000000
C	-1.3386000000	-18.8498000000	11.1858000000
N	-1.9433000000	-17.5376000000	11.1460000000
C	-0.8289000000	-16.6243000000	11.5470000000
C	2.3286000000	-19.4287000000	12.0146000000
C	1.0088000000	-19.8084000000	11.6706000000
C	0.0519000000	-18.8080000000	11.5574000000
C	0.3610000000	-17.5053000000	11.7627000000
N	1.6152000000	-17.1207000000	12.1059000000
C	-3.0800000000	-17.4750000000	12.0860000000
H	0.7397000000	-20.8405000000	11.4905000000
C	3.9918000000	-17.5799000000	12.6019000000
O	-1.9290000000	-19.8695000000	10.8625000000
H	3.6821000000	-23.6373000000	13.1592000000
H	-1.0325000000	-16.1031000000	12.5109000000
H	-2.1435000000	-16.3358000000	9.1458000000
H	-3.1401000000	-18.4250000000	12.6643000000
C	-4.4000000000	-17.3153000000	11.3188000000
H	-2.9509000000	-16.6441000000	12.8176000000
H	-4.4398000000	-18.0707000000	10.5099000000
C	-4.5219000000	-15.8976000000	10.7229000000
H	-5.2341000000	-17.5068000000	12.0259000000
H	-3.5157000000	-15.4240000000	10.6995000000
H	-5.1443000000	-15.2732000000	11.4008000000
H	-7.3136000000	-15.6812000000	7.7336000000
N	-5.1056000000	-15.8489000000	9.3641000000
C	-4.8589000000	-16.0902000000	6.9428000000
C	-4.1190000000	-16.0088000000	8.2832000000
C	-5.9587000000	-14.6728000000	9.1248000000

C	-6.6198000000	-14.8099000000	7.7478000000
O	-5.6408000000	-14.9281000000	6.7308000000
H	-7.2264000000	-13.9010000000	7.5505000000
H	-5.4907000000	-17.0066000000	6.9012000000
H	-4.1110000000	-16.1668000000	6.1257000000
H	-3.4204000000	-15.1445000000	8.2470000000
H	-3.5350000000	-16.9428000000	8.4332000000
H	-5.3638000000	-13.7341000000	9.1376000000
H	-6.7516000000	-14.6101000000	9.9016000000
C	-0.5669000000	-15.6194000000	10.4495000000
C	0.4576000000	-14.6678000000	10.5926000000
C	0.7002000000	-13.7330000000	9.5816000000
C	-0.0770000000	-13.7384000000	8.4192000000
C	-1.1000000000	-14.6794000000	8.2685000000
C	-1.3468000000	-15.6144000000	9.2778000000
H	1.0689000000	-14.6468000000	11.4861000000
H	1.4922000000	-13.0050000000	9.7033000000
Cl	0.2304000000	-12.5653000000	7.1520000000
H	-1.7049000000	-14.6870000000	7.3708000000
N	3.3891000000	-20.4021000000	12.1431000000
C	5.2299000000	-21.6260000000	11.0829000000
C	4.1120000000	-20.5971000000	10.8753000000
C	2.8946000000	-21.6827000000	12.6713000000
C	4.0687000000	-22.6593000000	12.8031000000
O	4.7010000000	-22.8531000000	11.5511000000
H	4.7961000000	-22.2931000000	13.5633000000
H	5.9992000000	-21.2327000000	11.7850000000
H	5.7340000000	-21.8063000000	10.1103000000
H	3.4316000000	-20.9634000000	10.0748000000
H	4.5632000000	-19.6352000000	10.5462000000
H	2.1278000000	-22.1271000000	11.9981000000
H	2.4415000000	-21.5259000000	13.6748000000
H	4.3365000000	-16.8432000000	11.8439000000
H	4.7027000000	-18.4341000000	12.5716000000
H	3.5905000000	-18.8617000000	14.9962000000
C	4.0143000000	-16.9906000000	13.9888000000
C	4.2627000000	-15.6190000000	14.1709000000
C	4.2993000000	-15.0745000000	15.4581000000
C	4.0820000000	-15.8915000000	16.5707000000
C	3.8232000000	-17.2538000000	16.3979000000
C	3.7849000000	-17.8028000000	15.1126000000
H	4.4349000000	-14.9755000000	13.3169000000
H	4.4974000000	-14.0191000000	15.5930000000
H	4.1127000000	-15.4689000000	17.5666000000
H	3.6554000000	-17.8857000000	17.2603000000

1k (enantiomer S)

C	2.09601680	1.26938621	0.32310825

C	-1.57265895	3.09349037	1.07135452
N	-2.39222513	2.00659053	1.55728413
C	-1.49314993	0.80262511	1.44123210
C	2.06847226	2.66804405	0.05711508
C	0.86353932	3.37873073	0.27284230
C	-0.23872039	2.66970203	0.73332784
C	-0.17210789	1.33879007	0.98060538
N	0.97154826	0.63813301	0.77776591
C	-3.69203368	1.96375007	0.85163904
H	0.78677690	4.44188079	0.08851643
C	3.33948300	0.45124203	0.09490328
O	-1.94215881	4.25824708	1.05278319
H	4.21557881	4.99363946	-3.23471217
H	-1.58881722	0.75501706	-1.34086639
H	5.81555379	1.46958537	-0.51172485
H	-4.13407535	2.98484766	0.81220161
C	-4.69855819	1.08497165	1.61032250
H	-3.57504627	1.65195046	-0.20403334
H	-4.20713616	0.16295702	1.96275087
C	-5.91355632	0.74151804	0.72247960
H	-5.03500336	1.64221880	2.51087757
H	-5.56084257	0.39487564	-0.27434748
H	-6.49475422	1.67145294	0.53948396
H	-8.17712651	-2.66445414	1.03332801
N	-6.81908946	-0.28230368	1.28906735
C	-8.35309893	-1.01544145	3.04873279
C	-7.33259516	0.04873037	2.62846690
C	-6.25595803	-1.64354048	1.26324987
C	-7.32141402	-2.63483723	1.74573372
O	-7.76211303	-2.30264041	3.05018257
H	-6.87708061	-3.65213457	1.77102061
H	-9.24327708	-0.98783682	2.37977238
H	-8.70486664	-0.78693659	4.07679838
H	-6.51141634	0.06587757	3.37863428
H	-7.82991215	1.04291984	2.61098605
H	-5.36928133	-1.72727068	1.92705455
H	-5.95587403	-1.91516368	0.22756821
C	4.44036323	0.84932243	1.04399246
C	4.26305449	0.71229779	2.43163925
C	5.27868682	1.09484213	3.31304791
C	6.47954133	1.60937723	2.81714607
C	6.66863277	1.73793287	1.43869953
C	5.65603583	1.35626517	0.55340990
H	3.33640965	0.31631692	2.82856051

H	5.13435910	0.99308185	4.38085507
H	7.26402762	1.90687220	3.50084116
H	7.59900434	2.13677899	1.05566280
N	3.26151279	3.32624542	-0.42491310
C	5.00442919	5.01006657	-0.05218180
C	3.73266749	4.36252346	0.50899855
C	3.07353749	3.87783724	-1.77551708
C	4.37746076	4.54054109	-2.23408097
O	4.76252262	5.56264042	-1.33309923
H	5.18421858	3.77967922	-2.34067094
H	5.83650468	4.27148555	-0.09025632
H	5.32049384	5.82616286	0.63101973
H	2.96341513	5.15256628	0.65699764
H	3.96629607	3.90370321	1.49489556
H	2.25770701	4.63463971	-1.79181668
H	2.81640736	3.05962478	-2.48362728
H	3.65751882	0.56997973	-0.96380676
H	3.11635876	-0.62709981	0.24771216
H	-1.34601564	0.39492097	2.46494334
C	-1.96025259	-0.31537346	0.51030515
C	-2.37489065	-1.54581345	1.05241417
C	-2.80722148	-2.58049866	0.21840358
C	-2.80850659	-2.40973647	-1.16836026
C	-2.36330504	-1.20660643	-1.72269469
C	-1.93166355	-0.16895397	-0.89201333
H	-2.38507934	-1.69828429	2.12441727
H	-3.14463801	-3.51319995	0.65218956
Cl	-3.36239468	-3.70612334	-2.21157516
H	-2.35205423	-1.07351850	-2.79692194

1l (enantiomer R)

C	3.2003000000	-13.9704000000	13.8079000000
C	2.5657000000	-14.8793000000	12.9708000000
C	1.2416000000	-14.7868000000	12.6989000000
N	0.4735000000	-13.8115000000	13.2446000000
C	1.0278000000	-12.8867000000	14.0857000000
C	2.4181000000	-12.9438000000	14.3890000000
C	3.0685000000	-16.0072000000	12.2294000000
C	0.8007000000	-15.8694000000	11.7634000000
C	0.1434000000	-11.8100000000	14.6569000000
H	4.4037000000	-11.9256000000	18.3762000000
H	4.2614000000	-14.0578000000	13.9994000000
O	4.2498000000	-16.3148000000	12.1808000000
N	2.0338000000	-16.6768000000	11.4691000000

H	0.0335000000	-16.4682000000	12.3078000000
H	1.9194000000	-14.0734000000	9.9899000000
C	1.8607000000	-18.0857000000	11.8938000000
H	-0.2401000000	-17.7832000000	14.2386000000
N	2.9962000000	-11.9585000000	15.2743000000
C	4.6451000000	-10.1806000000	15.6108000000
C	4.0926000000	-11.2202000000	14.6290000000
C	3.4367000000	-12.5546000000	16.5466000000
C	4.0249000000	-11.4583000000	17.4431000000
O	5.1021000000	-10.8058000000	16.7961000000
H	3.2380000000	-10.7275000000	17.7364000000
H	3.8719000000	-9.4118000000	15.8394000000
H	5.5008000000	-9.6580000000	15.1341000000
H	4.9158000000	-11.9048000000	14.3257000000
H	3.7121000000	-10.6960000000	13.7250000000
H	4.2115000000	-13.3354000000	16.3798000000
H	2.5695000000	-13.0186000000	17.0660000000
H	0.6738000000	-10.8354000000	14.5865000000
H	0.7780000000	-14.0262000000	16.1474000000
H	-0.7834000000	-11.7198000000	14.0496000000
C	-0.2470000000	-12.1182000000	16.0795000000
C	-1.0316000000	-11.2099000000	16.8111000000
C	-1.4061000000	-11.5020000000	18.1260000000
C	-0.9953000000	-12.6972000000	18.7224000000
C	-0.2053000000	-13.6001000000	18.0062000000
C	0.1721000000	-13.3122000000	16.6912000000
H	-1.3578000000	-10.2810000000	16.3597000000
H	-2.0157000000	-10.8020000000	18.6826000000
H	-1.2878000000	-12.9226000000	19.7397000000
H	0.1121000000	-14.5257000000	18.4689000000
C	0.1929000000	-15.2807000000	10.5029000000
C	-1.1070000000	-15.6453000000	10.1105000000
C	-1.6716000000	-15.1080000000	8.9497000000
C	-0.9459000000	-14.2035000000	8.1705000000
C	0.3468000000	-13.8347000000	8.5501000000
C	0.9162000000	-14.3693000000	9.7094000000
H	-1.6839000000	-16.3466000000	10.7003000000
H	-2.6728000000	-15.3938000000	8.6536000000
H	-1.3852000000	-13.7890000000	7.2724000000
H	0.9084000000	-13.1342000000	7.9455000000
H	2.1181000000	-18.2066000000	12.9712000000
C	2.7046000000	-19.0663000000	11.0551000000
H	0.7895000000	-18.3650000000	11.7836000000
H	2.0566000000	-19.6713000000	10.3805000000
N	3.5000000000	-19.9418000000	11.9442000000
H	3.3744000000	-18.4610000000	10.4059000000
C	4.3868000000	-20.8102000000	11.1491000000
C	2.6301000000	-20.7553000000	12.8261000000

H	1.6064000000	-20.3222000000	12.8937000000
C	3.2079000000	-20.7888000000	14.2455000000
H	2.5072000000	-21.7906000000	12.4329000000
H	5.1298000000	-20.1926000000	10.6002000000
H	3.8050000000	-21.4147000000	10.4178000000
H	4.9603000000	-21.4950000000	11.8085000000
H	4.2745000000	-21.0994000000	14.1869000000
H	2.6775000000	-21.5445000000	14.8628000000
H	5.2228000000	-19.0804000000	14.9792000000
C	3.0900000000	-19.4414000000	14.9105000000
C	1.8259000000	-18.9127000000	15.2150000000
C	1.7067000000	-17.6616000000	15.8454000000
C	2.8614000000	-16.9223000000	16.1499000000
C	4.1255000000	-17.4420000000	15.8204000000
C	4.2388000000	-18.6982000000	15.2209000000
H	0.9332000000	-19.4685000000	14.9556000000
O	0.4633000000	-17.1104000000	16.0923000000
O	2.7312000000	-15.6548000000	16.6879000000
H	5.0208000000	-16.8664000000	16.0165000000
C	4.0107000000	-15.0762000000	16.8195000000
C	-0.1780000000	-16.8615000000	14.8613000000
H	4.7015000000	-15.7329000000	17.3963000000
H	3.9105000000	-14.1253000000	17.3819000000
H	4.4468000000	-14.8308000000	15.8269000000
H	0.3284000000	-16.0471000000	14.2986000000
H	-1.2160000000	-16.5285000000	15.0661000000

1l (enantiomer S)

C	3.66300631	0.30055879	-1.25824160
C	2.39963194	0.46416602	-0.70417736
C	2.10239264	-0.00334347	0.53239889
N	3.02224465	-0.66392736	1.27871519
C	4.28256289	-0.87422085	0.79250136
C	4.63671833	-0.39197517	-0.49955501
C	1.21034681	1.09810874	-1.21048818
C	0.66590184	0.24259422	0.87705494
C	5.26855911	-1.62452747	1.64751503
H	7.38074193	-2.22888301	-3.65750248
H	3.87799762	0.68973963	-2.24438045
O	1.16412480	1.70382006	-2.27012391
N	0.11098280	1.04331205	-0.27059415
H	0.65032287	-2.08979556	-0.63044845
H	-7.22735128	1.56970008	-0.28422443
C	-1.12765988	0.52331129	-0.89371029
H	-10.23642216	0.34533557	1.05046586
N	5.97079597	-0.62190731	-1.00572249

C	8.10924761	0.32301678	-1.73165354
C	6.69991654	0.63793912	-1.21733789
C	5.96008718	-1.43859579	-2.23085630
C	7.40160371	-1.65850933	-2.70518613
O	8.04792595	-0.41999191	-2.93539228
H	7.96815551	-2.26985747	-1.96710632
H	8.69333395	-0.22364258	-0.95633573
H	8.63836818	1.27829837	-1.93224205
H	6.18006371	1.28476256	-1.95895005
H	6.78215943	1.19110760	-0.25597550
H	5.38729447	-0.93961099	-3.04374552
H	5.49408567	-2.42662895	-2.02184736
H	6.17687984	-0.99814789	1.78438645
H	7.72170502	-2.53015373	0.81239004
H	4.83817432	-1.79829808	2.65770855
C	5.61005819	-2.96025326	1.03920998
C	4.60653551	-3.92431186	0.83907875
C	4.91945446	-5.15689127	0.25829547
C	6.23473681	-5.43915330	-0.12005251
C	7.24028977	-4.49099016	0.08593984
C	6.93235448	-3.25721218	0.66704777
H	3.58262756	-3.71668139	1.12528088
H	4.14176007	-5.89286105	0.10032135
H	6.47496680	-6.39297359	-0.57158314
H	8.25852583	-4.71047039	-0.20799797
O	-8.88329054	1.76142610	1.79346085
O	-8.30770878	0.04189946	3.87345780
H	-6.18415036	-1.52437100	3.45023490
C	-9.07897573	-1.13447813	3.97344824
C	-9.91860026	1.40323126	0.90525508
H	-9.69602298	-1.29911411	3.06060707
H	-9.77565140	-1.02672519	4.82986354
H	-8.43912224	-2.02123816	4.17427293
H	-9.62240510	1.57764644	-0.15200871
H	-10.79865386	2.04563003	1.11350993
H	-0.90854688	-0.30100426	-1.60964136
C	-1.89014319	1.64574945	-1.61451184
H	-1.80464042	0.12391044	-0.11176002
H	-1.94853622	2.55207169	-0.96916174
N	-3.24171281	1.17908785	-1.99286262
H	-1.30963842	1.92642551	-2.51988710
C	-3.73627545	1.91310769	-3.16963070
C	-4.20209347	1.28151046	-0.86795678
H	-3.68196679	1.38610684	0.11123004

C	-5.05660467	0.01241224	-0.81258323
H	-4.85703670	2.17812672	-0.96302853
H	-3.08939874	1.70306499	-4.04825196
H	-3.74955106	3.00957417	-2.97959153
H	-4.76258716	1.57668464	-3.43178116
H	-4.38146227	-0.87197305	-0.81569815
H	-5.69595916	-0.05590257	-1.71910438
H	-4.79377652	-1.54450253	1.42801728
C	-5.92773710	-0.00223139	0.41680709
C	-7.02086203	0.87228668	0.51817061
C	-7.84281221	0.86057372	1.65937859
C	-7.55019141	-0.01455065	2.71827232
C	-6.43936925	-0.87083240	2.62595956
C	-5.64429761	-0.87546836	1.47797033
H	0.64233148	0.88620674	1.78312932
C	-0.03300226	-1.08269274	1.16643436
C	-0.80180648	-1.22623811	2.33518954
C	-1.46609046	-2.42706610	2.60269162
C	-1.36436472	-3.49821298	1.71199567
C	-0.59782676	-3.37135582	0.55136014
C	0.06518187	-2.17204304	0.27688763
H	-0.89805044	-0.40502800	3.03430729
H	-2.06189213	-2.52644651	3.50082029
H	-1.87960731	-4.42670547	1.92130645
H	-0.51922734	-4.20213078	-0.13786571

Paclitaxel

C	-2.84343480	1.65022796	-0.13091776
C	-2.43592671	3.28111717	1.78359406
C	-3.20347551	2.87480336	0.71589175
C	-1.44772985	1.01044599	0.16192548
C	-0.58391905	1.65893497	1.30318156
C	-1.53698323	2.20753854	2.43491394
H	-3.64835712	0.91295208	0.09711626
O	-2.89585712	2.02898203	-1.50034282
C	-2.39634458	4.78371078	2.12349291
C	-0.11970182	3.93017032	-0.23204150
C	-4.38527758	3.68492068	0.22003292
H	-0.85162281	0.96326320	-0.77557579
H	-1.63871620	-0.05928702	0.39639863
C	0.44275164	2.71216629	0.67399096
O	0.24247586	0.64835549	1.85643754
H	1.20140022	0.58813996	-4.75955528

H	-5.00526990	4.05191111	1.06299610
H	-5.05501166	3.04914684	-0.39649770
H	-4.04229359	4.52745307	-0.41229828
C	-3.17052894	0.96782353	-2.36829543
C	2.68958200	3.28071029	1.18665647
H	-1.14115637	3.61073847	-0.41566410
C	1.20003629	2.24472424	-3.37871239
H	0.06018383	0.43904449	-3.36585360
H	0.98074269	2.00319500	0.02157082
O	1.38153393	3.11586269	1.65686629
O	-3.46613091	0.07789967	-4.58444495
C	1.06858697	0.78595925	-3.67441553
O	1.45514003	3.02047747	-4.28921324
H	1.83726099	0.22222851	-3.10614275
O	-1.72405601	5.05980351	3.33629248
H	-3.46202074	5.07792206	2.24034894
C	-1.78168610	5.57186184	0.96351162
C	-2.20934341	6.17145557	4.02454486
O	-3.26896992	-0.19030602	-1.97617633
C	-3.30320520	1.26628180	-3.84604239
H	-2.34640195	1.72525483	-4.16750132
C	-4.46365984	2.25832870	-4.12751348
H	-0.30560080	-0.13157243	2.12668277
O	-2.13974971	6.16281243	5.24315754
C	-2.69872253	7.43213823	3.35293393
H	-2.89253263	8.22191664	4.11001642
H	-3.64664853	7.25318740	2.80665994
H	-1.92291358	7.81261804	2.65736390
C	-0.32277294	5.40734981	0.43434050
O	-2.51817790	6.38973480	0.42680434
C	-0.07912964	6.53524194	-0.64274489
C	0.63785988	5.74786577	1.61162025
H	-1.63004986	4.64052628	-2.33359766
H	0.50954987	5.10620580	2.47952115
H	0.44013416	6.76250164	2.01638019
H	1.70192433	5.71623370	1.31841193
O	-0.05364617	7.81422565	-0.05109592
H	-0.91934223	6.53242076	-1.37182199
C	1.21928570	6.31216409	-1.42431812
H	-0.20687560	8.47873932	-0.77341742
H	2.09501757	6.18358228	-0.75943162
H	1.42741485	7.20300847	-2.05636044
C	1.09652238	5.10621935	-2.34157482
O	0.07737310	5.32159777	-3.29813999

C	0.40057890	3.87798581	-1.75762967
H	2.07107372	4.88973847	-2.83038300
C	-0.68794506	4.25742825	-2.78122841
H	-4.18199458	7.59415126	-6.57170346
O	1.10860474	2.66146252	-2.05131574
H	-0.92669676	3.51095271	-3.56302832
C	-0.73342844	2.61956420	3.70296623
C	-2.49331571	1.09853600	2.99704835
H	-0.15622860	1.75730583	4.10083277
H	-1.41476072	2.94318084	4.52001170
H	0.00318070	3.40593202	3.54385600
H	-1.94758205	0.33354648	3.58984693
H	-3.06094171	0.54694322	2.22918364
H	-3.24504859	1.55634651	3.67723509
O	2.95238164	3.29934112	-0.00999857
H	5.35266565	3.71884912	0.69113662
C	3.77758922	3.50012129	2.17085270
C	3.49747981	3.50360156	3.55000794
C	4.51991720	3.71643296	4.47965618
C	5.83017933	3.92745898	4.04505756
C	6.12116923	3.92609865	2.67920764
C	5.10280825	3.71403257	1.74474637
H	2.49090980	3.34220886	3.91295348
H	4.29559605	3.71768133	5.53854651
H	6.61993955	4.09205987	4.76662919
H	7.13704968	4.09006513	2.34354791
H	-4.35510222	-0.30095556	-4.35385089
N	-4.20341175	3.55090232	-3.46499104
H	-5.37594677	1.80102397	-3.67766320
H	-2.65560917	3.07345275	-6.06921563
C	-4.68508591	2.44431961	-5.62242893
C	-5.95314741	2.19863049	-6.17930205
C	-6.17230499	2.37814447	-7.54830469
C	-5.12987864	2.80203653	-8.37544355
C	-3.86544367	3.04639643	-7.83541365
C	-3.64057044	2.86785406	-6.46747687
H	-6.77568784	1.87469840	-5.55403061
H	-7.15210652	2.18997485	-7.96786229
H	-5.30219506	2.94200289	-9.43473942
H	-3.05893733	3.37748742	-8.47676528
C	-5.39654695	4.36471770	-3.38926400
H	-3.41425917	4.05209892	-3.92656602
H	-3.94324244	5.39725174	-5.54359573
O	-6.32929733	3.96955521	-2.70390174

C	-5.50585135	5.69862552	-4.05405516
C	-6.47322486	6.61937103	-3.60246989
C	-6.60322006	7.87291067	-4.20722101
C	-5.77571434	8.22491082	-5.27384198
C	-4.81829386	7.32282836	-5.73908489
C	-4.68208571	6.06803027	-5.13748049
H	-7.12552183	6.37445233	-2.77340978
H	-7.34704549	8.57212064	-3.84746876
H	-5.87848116	9.19533404	-5.74201503

5. References

[S1] Zamudio-Medina, A.; García-González, M.C.; Gutierrez-Carrillo, A.; González-Zamora, E. Synthesis of cyclic analogues of hexamethylenebis(3-pyridine)amide (HMBPA) in a one-pot process. *Tetrahedron Lett.* **2015**, *56*, 627–629, DOI: 10.1016/j.tetlet.2014.12.018

[S2] Zamudio-Medina, A.; García-González, A.N.; Herrera-Carrillo, G.K.; Zárate-Zárate, D.; Benavides-Macías, A.; Tamariz, J.; Ibarra, I.A.; Islas-Jácome, A.; González-Zamora, E. *Molecules* **2018**, *23*, 763, DOI: 10.3390/molecules23040763
